# Supplementary figures and images for: Maternal circulating miRNAs contribute to negative pregnancy outcomes by altering placental transcriptome and fetal vascular dynamics
Source: PLoS One. 2023 Nov 6;18(11):e0290720. doi: 10.1371/journal.pone.0290720 (PMC10627460; doi:10.1371/journal.pone.0290720)

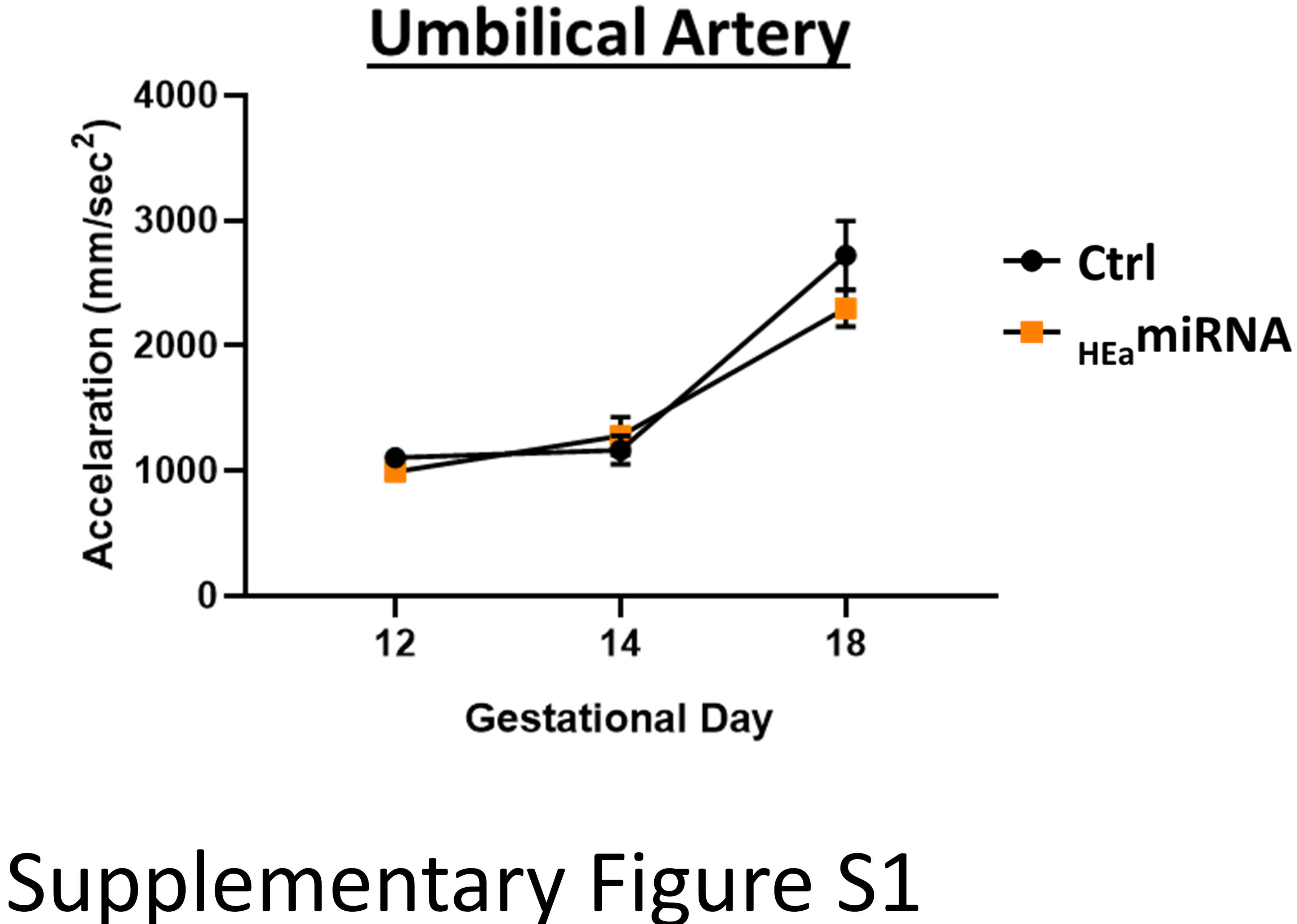

Supplement: S1 Fig — Results are expressed as the mean ± SEM, Control n = 10, mHEamiRNA n = 12. (TIF) [file pone.0290720.s001.tif]

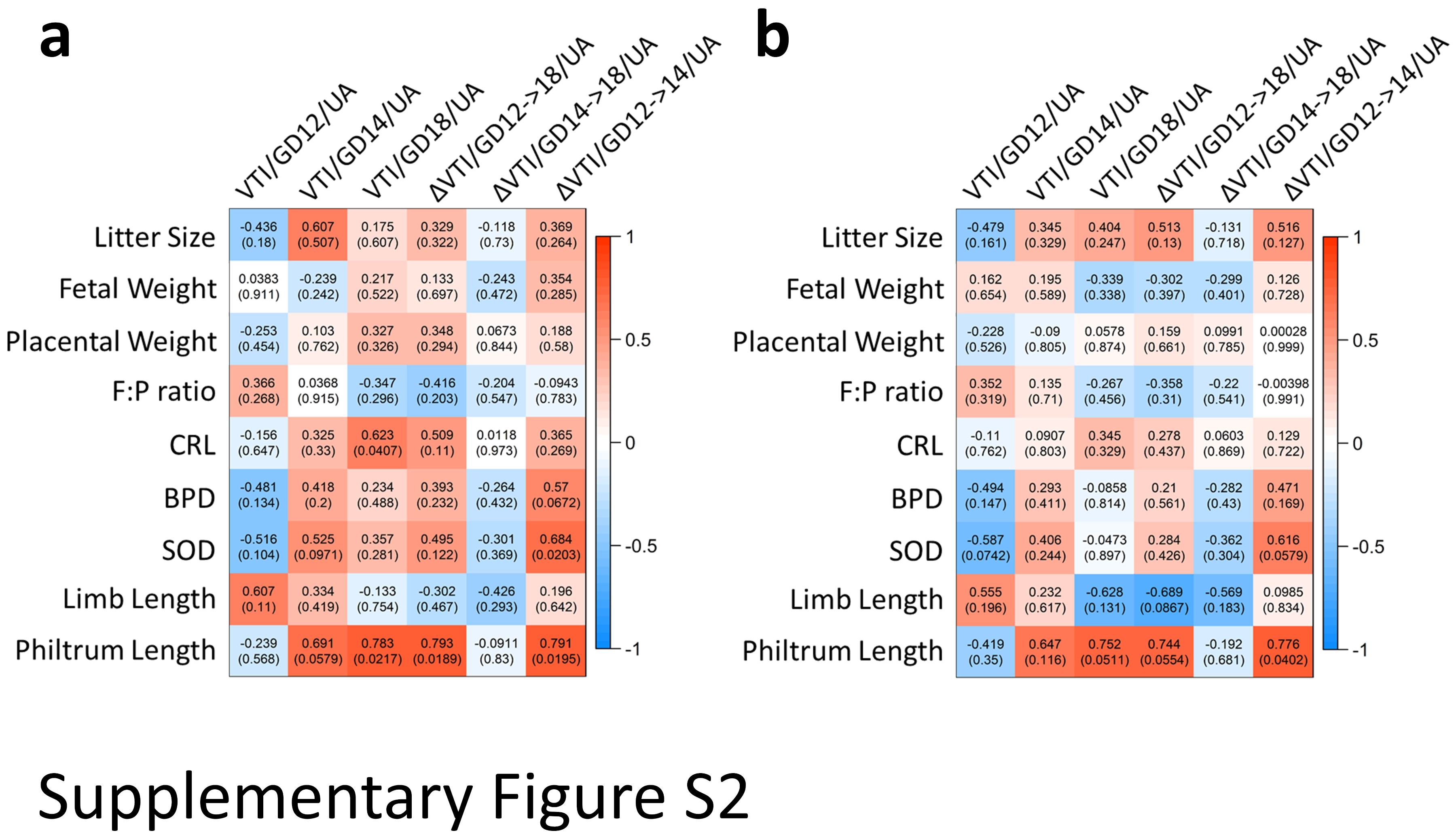

Supplement: S2 Fig — Heatmap of Pearson correlation matrices for uncorrected analyses (a) and analyses correcting for prenatal mHEamiRNA exposure (b). For all correlation matrices, color intensity indicates a stronger correlation, with red representing a positive relationship and blue representing a negative relationship. BPD = biparietal diameter. CRL = crown-rump length. F:P ratio = placental efficiency. SOD = snout-occipital distance. VTI = Velocity Time Integral. UA = umbilical artery. Control male n = 4, Control female n = 5, mHEamiRNA male n = 6, mHEamiRNA female n = 4. (TIF) [file pone.0290720.s002.tif]

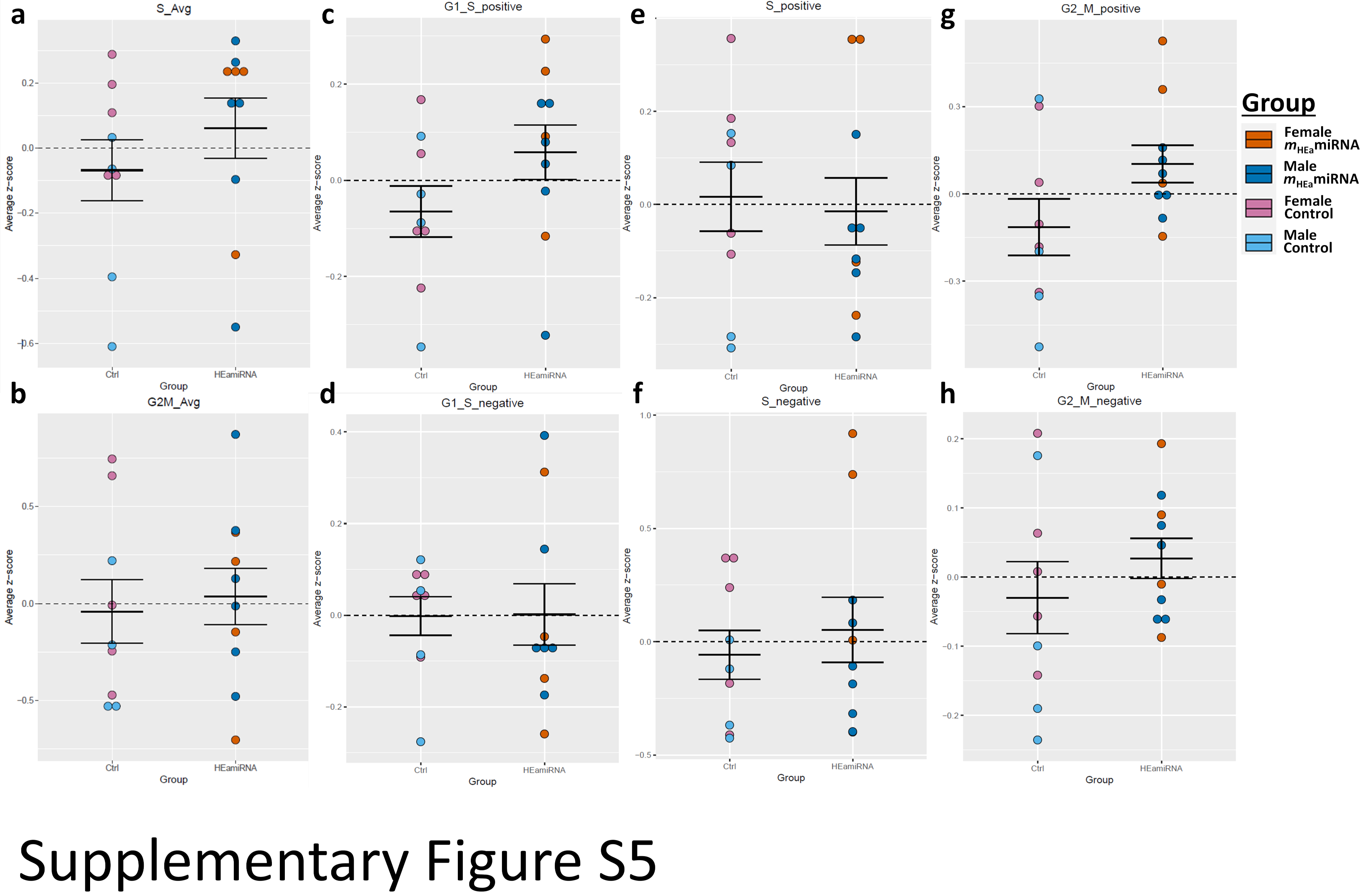

Supplement: S3 Fig — Quantification by average z-score of S-phase-associated genes (a) and of G2/M-phase-associated genes (b). Individual genes were z-scored across samples, and then, average z-score for each sample was calculated and used for analysis. Quantification by average z-score of G1-S transition-positive regulators (c), G1-S transition-negative regulators (d), S-phase-positive regulators (e), S-phase-negative regulators (f), G2-M transition-positive regulators (g), and G2-M transition-negative regulators (h). Control male n = 4, Control female n = 5, mHEamiRNA male n = 6, mHEamiRNA female n = 4. (TIF) [file pone.0290720.s003.tif]

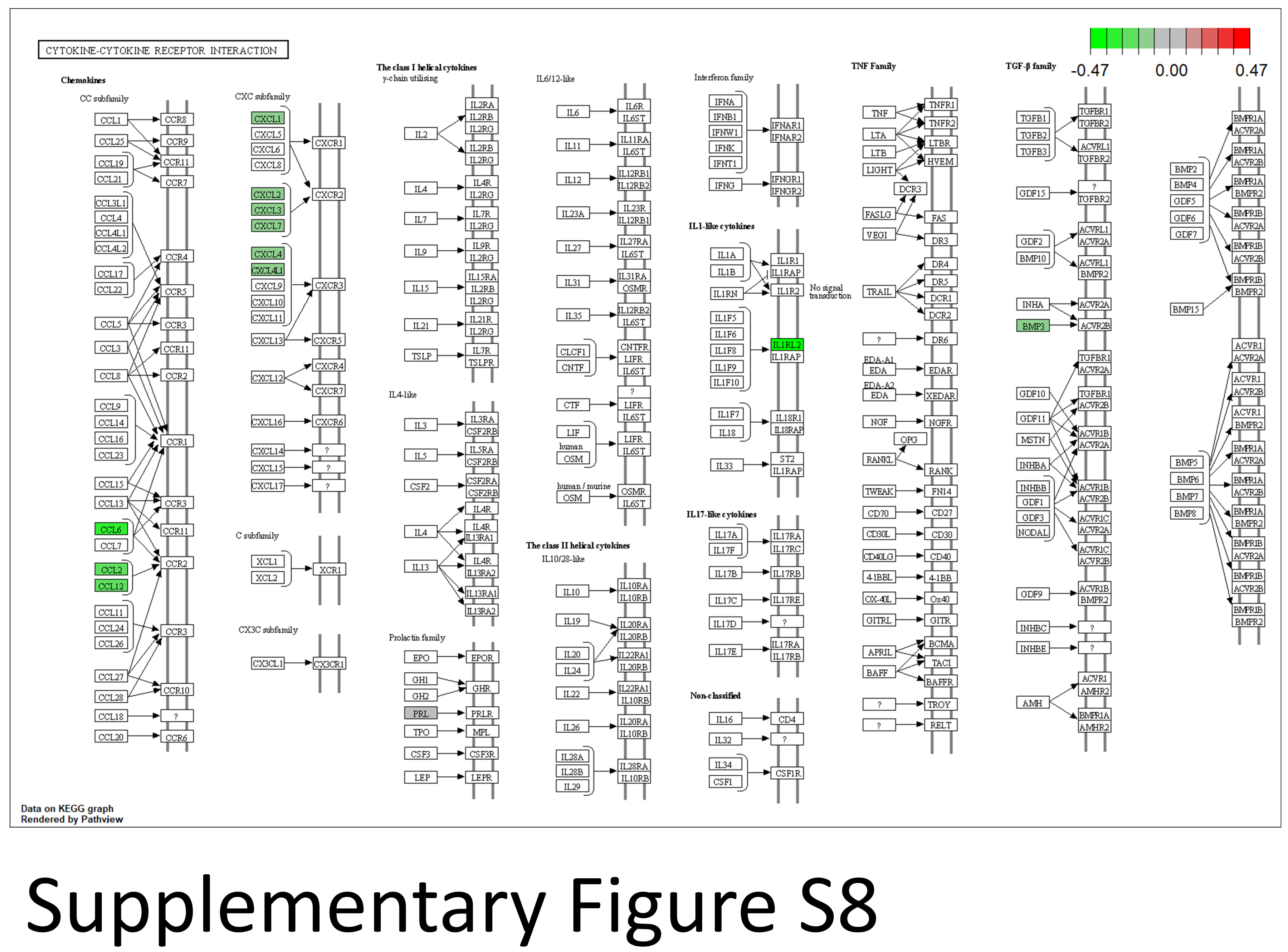

Supplement: S4 Fig — Control male n = 4, Control female n = 5, mHEamiRNA male n = 6, mHEamiRNA female n = 4. (TIF) [file pone.0290720.s004.tif]

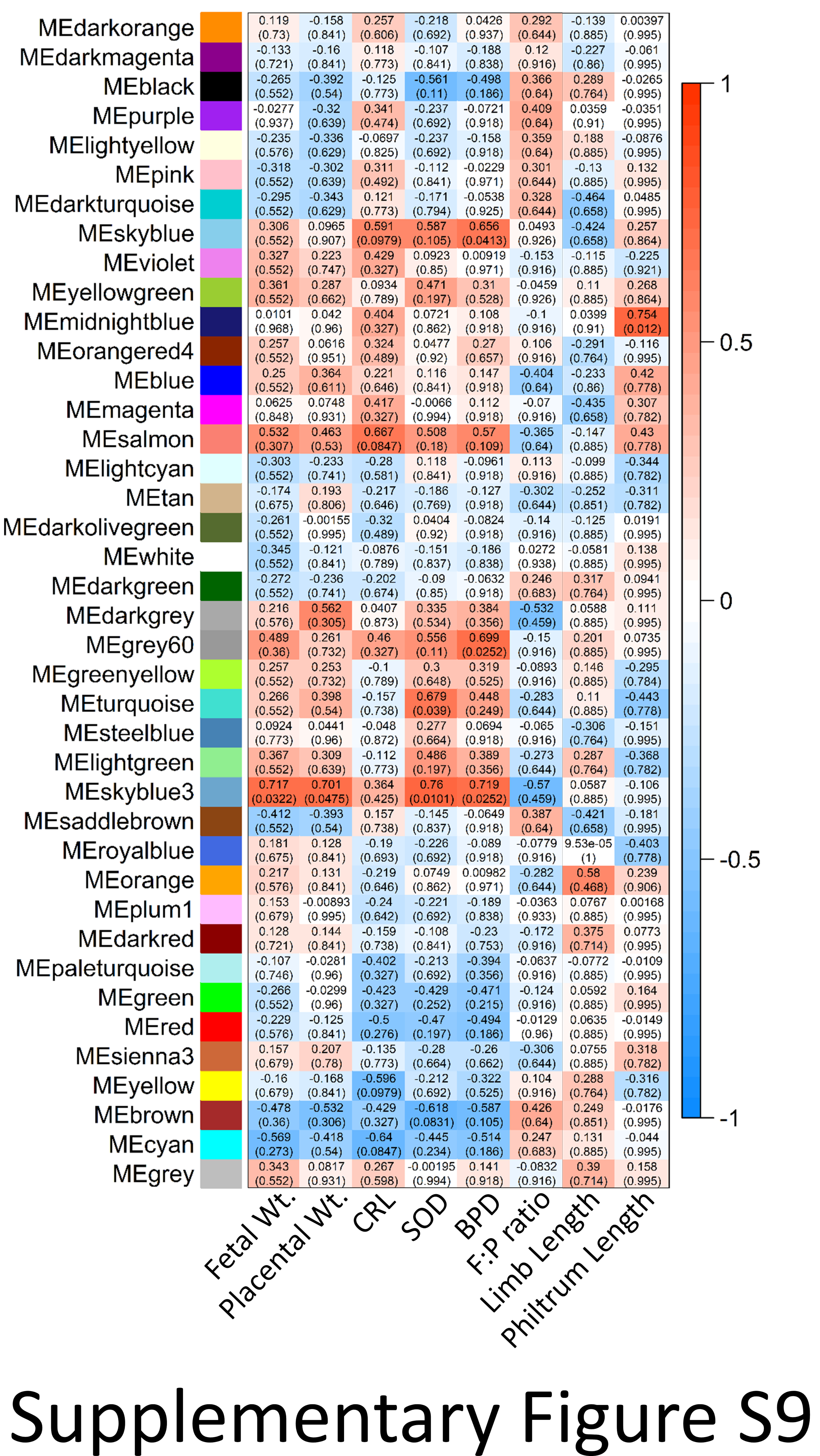

Supplement: S5 Fig — The degree of correlation (r) is illustrated with the color legend and is listed at the top of each square. P-values are Benjamini-Hochberg adjusted and are denoted in parenthesis in each square. Control male n = 4, Control female n = 5, mHEamiRNA male n = 6, mHEamiRNA female n = 4. (TIF) [file pone.0290720.s005.tif]

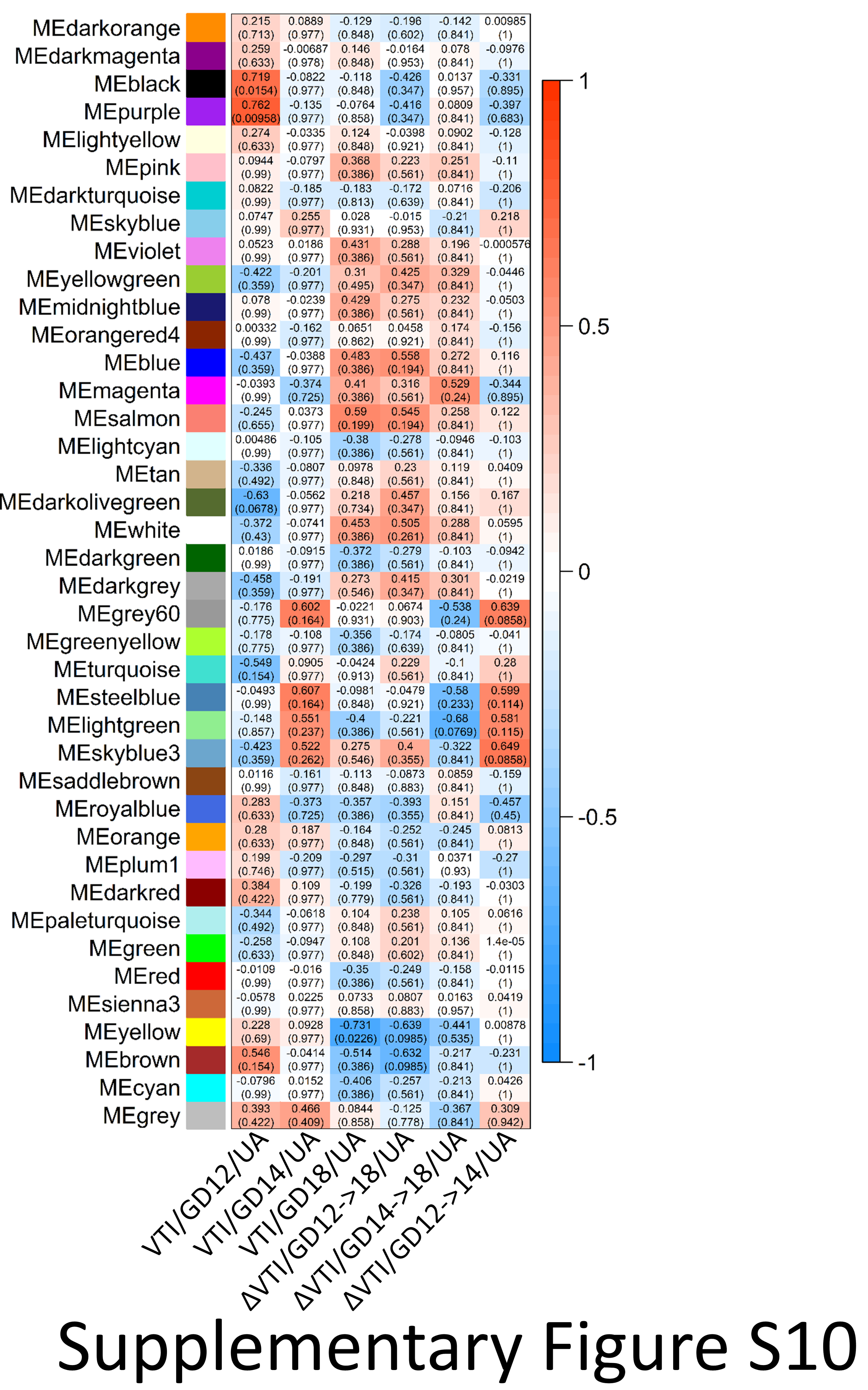

Supplement: S6 Fig — The degree of correlation (r) is illustrated with the color legend and is listed at the top of each square. P-values are Benjamini-Hochberg adjusted and are denoted in parenthesis in each square. Control male n = 4, Control female n = 5, mHEamiRNA male n = 6, mHEamiRNA female n = 4. (TIF) [file pone.0290720.s006.tif]

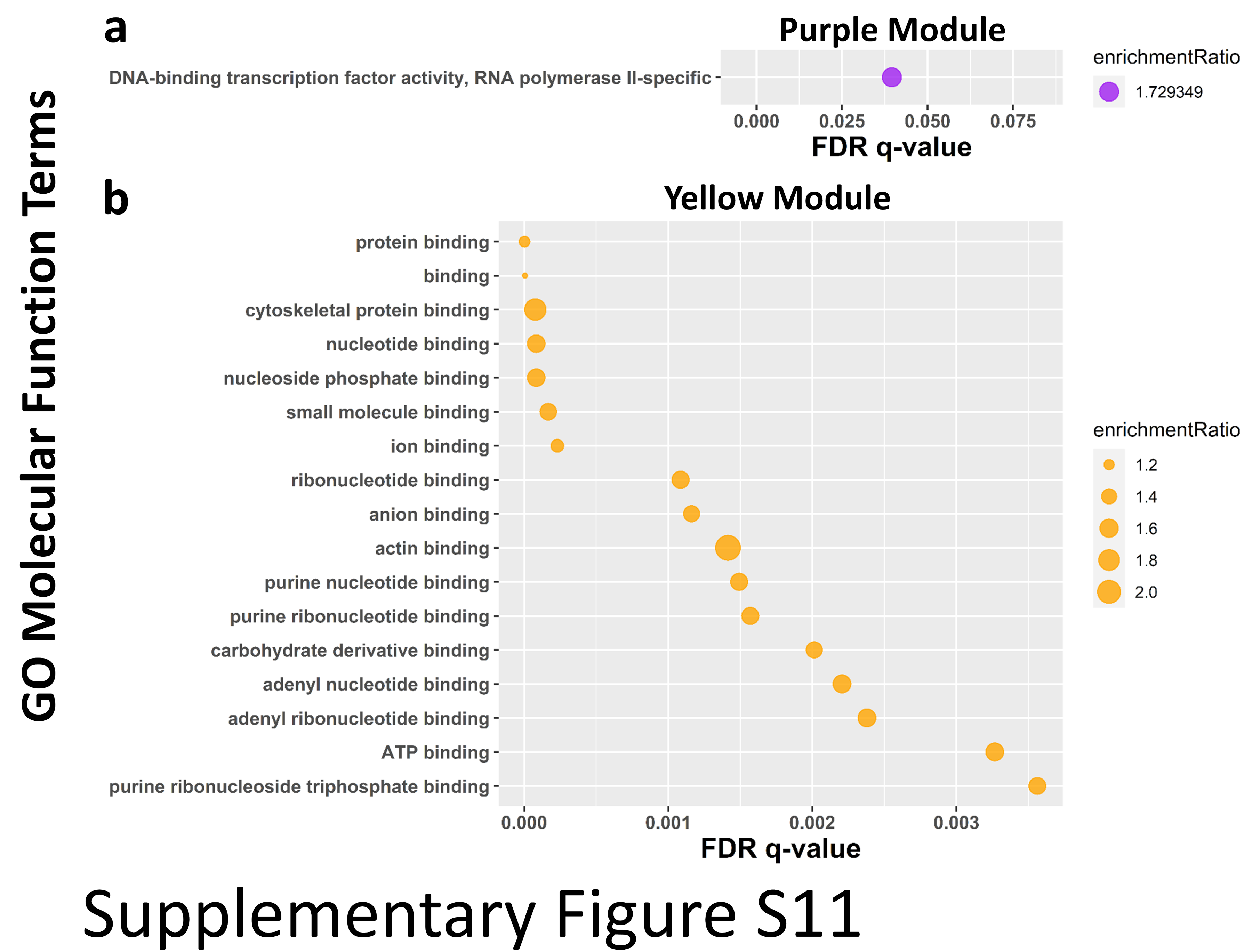

Supplement: S7 Fig — Gene ontology (GO) analysis of purple (a) and yellow (b) module genes focused on molecular function. Control male n = 4, Control female n = 5, mHEamiRNA male n = 6, mHEamiRNA female n = 4. (TIF) [file pone.0290720.s007.tif]
